# Prescribed Medication Burden by Tobacco Smoking and E‐Cigarette Use Status

**DOI:** 10.1002/hsr2.72072

**Published:** 2026-03-09

**Authors:** Yusuff Adebayo Adebisi, Leonard Ighodalo Uzairue, Rachael Oluwatoyosi Farayola, Eseosa Iyagbaye, Christian Joseph N. Ong, Akpevwe Emmanuella Benson, Ngoni Veddie Muzondo, Adeyemo Deborah Temitope

**Affiliations:** ^1^ College of Social Sciences University of Glasgow Glasgow UK; ^2^ Faculty of Health and Life Sciences De Montfort University, The Gateway Leicester UK; ^3^ Centre for Lifestyle Medicine and Behaviour, School of Sport Exercise and Health Sciences Loughborough University Loughborough UK; ^4^ Chronic Disease Mate for Africa (CDMA) Benin City Nigeria; ^5^ Department of Biology, College of Science De La Salle University Manila Philippines; ^6^ Faculty of Pharmacy University of Benin Benin City Edo Nigeria; ^7^ Department of Pharmaceutical Technology Harare Institute of Technology Harare Zimbabwe; ^8^ Drug and Toxicological Information Services Harare Zimbabwe; ^9^ Generational Stewards for Antimicrobials (GSA) Harare Zimbabwe; ^10^ Faculty of Pharmaceutical Sciences University of Ilorin Ilorin Nigeria

**Keywords:** e‐cigarette use, ex‐smokers, Health Survey for England, medication burden, prescribing patterns, public health, Tobacco smoking

## Abstract

**Purpose:**

Tobacco smoking is a major driver of chronic disease, and e‐cigarette use is increasingly common, yet their associations with prescribed medication burden remain unclear.

**Methods:**

We analysed data from 1657 adults aged ≥ 16 years who completed a nurse visit in the Health Survey for England 2021. Smoking and e‐cigarette status were categorized into four mutually exclusive groups: never users, current smokers, current exclusive e‐cigarette users, and ex‐smokers. Prescribed medication use in the past 7 days was verified by nurses and grouped into seven BNF‐based therapeutic categories: cardiovascular, metabolic, respiratory, pain and anti‐inflammatory, gastrointestinal, mental health, and anti‐infectives. Overall, medication burden was categorized as none, one, two, or three or more prescribed medicines. Generalized ordered logistic regression estimated associations with overall burden, and Firth penalized logistic regression examined therapeutic categories, adjusting for age, sex, education, and area‐level deprivation. Sensitivity analyses restricted the sample to adults aged ≥ 45 years.

**Results:**

Ex‐smokers reported the highest medication burden, with 20.3% prescribed three or more medicines compared with 8.6% of never users, 10.9% of current smokers, and 12.2% of current e‐cigarette users (*p* < 0.001). In adjusted analyses using never users as the reference group, ex‐smokers had significantly greater odds of higher overall burden (OR = 1.50, 95% CI: 1.21–1.87, *p* < 0.001). Current smokers (OR = 0.86, 95% CI: 0.60–1.24, *p* = 0.42) and e‐cigarette users (OR = 1.63, 95% CI: 0.93–2.83, *p* = 0.09) did not differ significantly from never users. By therapeutic category, current e‐cigarette users, compared with never users, had higher odds of pain and anti‐inflammatory medication use (OR = 3.61, 95% CI: 1.70–7.67, *p* = 0.001). Ex‐smokers, relative to never users, also had elevated odds of respiratory (OR = 1.72, 95% CI: 1.17–2.55, *p* = 0.006), gastrointestinal (OR = 1.56, 95% CI: 1.17–2.07, *p* = 0.002), and anti‐infective prescriptions (OR = 3.32, 95% CI: 1.63–6.79, *p* = 0.001). Sensitivity analyses restricting to those aged ≥ 45 years produced consistent results.

**Conclusions:**

Ex‐smokers had the highest medication burden compared with never users, which may partly reflect reverse causation, whereby illness and associated treatment prompt smoking cessation, though residual effects of prior smoking cannot be excluded. Current smokers showed no significant excess burden, possibly due to a “healthy smoker” effect, and e‐cigarette use was not associated with overall burden compared with never users, although it was linked to greater use of pain and anti‐inflammatory medicines.

## 1 Introduction

Medication burden, defined as the number of prescribed medicines an individual is taking, has become a key marker of population health and healthcare utilization [1, 2, 3]. While many prescriptions are clinically necessary, a higher medication burden is associated with several challenges, including difficulties in adherence, heightened risk of drug–drug interactions, and increased likelihood of adverse events, all of which contribute to avoidable morbidity and greater strain on health services [4, 5, 6, 7]. At a population level, medication burden is influenced not only by the prevalence of chronic conditions but also by prescribing practices, access to healthcare, and sociodemographic factors such as age, sex, and deprivation [8, 9]. Examining differences in medication burden between groups with distinct health behaviors can therefore provide important insights into the long‐term consequences of those behaviors, as well as their contribution to health inequalities and healthcare utilization.

Tobacco smoking is one of the strongest behavioral determinants of chronic disease and, consequently, of medication use [10, 11, 12]. Smokers face a significantly higher risk of cardiovascular disease, diabetes, respiratory conditions such as asthma and chronic obstructive pulmonary disease (COPD), and gastrointestinal disorders, all of which often require long‐term pharmacological management [13, 14]. Smoking cessation reduces disease risk, yet former smokers may continue to exhibit a higher medication burden because many smoking‐related illnesses persist after quitting [15, 16]. This suggests that smoking status is not only relevant for immediate health outcomes but also for longer‐term prescribing patterns, with implications for how we understand the enduring effects of tobacco use on healthcare systems.

The increasing popularity of e‐cigarettes introduces a further dimension to this issue. E‐cigarettes are widely used in England, often by people who currently smoke or have recently quit [17, 18]. However, their relationship with prescribed medication burden is poorly understood. Most existing research has examined outcomes such as disease incidence or health assessment [19, 20, 21, 22], while prescribing patterns among e‐cigarette users have received little attention. It remains unclear whether exclusive e‐cigarette users resemble smokers, ex‐smokers, or never users in terms of their medication profiles. Addressing this gap is important given ongoing debates about the role of e‐cigarettes in public health and the growing number of adults reporting regular use.

Medication burden also reflects broader social and economic patterns. Both smoking prevalence and prescription use are socially stratified, with higher levels observed among people living in more deprived areas [8, 9]. Deprivation influences not only smoking behaviors but also the development of chronic conditions and access to healthcare, leading to systematic differences in prescribing [9]. These inequalities are likely to be compounded by educational attainment, sex, and age, all of which shape health trajectories and healthcare interactions [8, 23, 24]. Disentangling the contribution of smoking and e‐cigarette status from these background sociodemographic influences is therefore crucial for producing valid evidence. Such analyses can clarify whether observed differences in medication burden are attributable to tobacco and nicotine behaviors themselves, or whether they primarily reflect the social patterning of health and healthcare use.

To our knowledge, this is the first study to examine differences in prescribed medication burden by smoking and e‐cigarette status in a nationally representative sample of adults in England. We analysed both prescriptions across major therapeutic categories and the total number of prescribed medications, adjusting for sociodemographic factors.

## 2 Methods

### 2.1 Study Design and Data Source

This study used a cross‐sectional design to examine differences in prescribed medication burden by smoking and e‐cigarette status. Data were drawn from the Health Survey for England (HSE) 2021, an annual, nationally representative survey of individuals living in private households across England. The HSE is commissioned by NHS England and conducted by the National Centre for Social Research (NatCen) in collaboration with University College London (UCL). Due to COVID‐19 restrictions, the 2021 survey employed a mixed‐mode approach: Stage 1 household interviews were conducted remotely by telephone or video, while Stage 2 nurse visits (resumed in October 2021) collected physical measurements, biological samples, and detailed information on medication use [25].

A stratified, multistage probability sampling design was used, with 12,798 addresses selected across 711 postcode sectors [25]. Of these, 5880 adults aged 16 years and over and 1240 children were interviewed, with 1705 adults completing a nurse visit. Response rates and fieldwork procedures are described in detail elsewhere. This analysis was restricted to adults ( ≥ 16 years) who completed a nurse visit, as only this subsample provided verified information on prescribed medications. All 1705 adults who attended a nurse visit were eligible for participation. After excluding participants with missing information on smoking or e‐cigarette use, the final analytic sample comprised 1657 adults.

### 2.2 Outcomes

Prescribed medication use was ascertained during the nurse visit using the Health Survey for England prescribed medicines module. Participants were asked whether they were taking or using any medicines prescribed for them by a doctor or nurse. Nurses recorded the names of prescribed medicines (requesting to see medication containers where possible) and asked whether each medicine had been taken or used in the past 7 days. This time frame minimizes recall bias and captures current prescribing patterns [8]. Individual variables were initially coded for specific drug classes (e.g., antihypertensives, lipid‐lowering agents, antidepressants, proton pump inhibitors). These detailed drug indicators were then collapsed into clinically meaningful therapeutic categories using British National Formulary groupings [26]. Specifically, cardiovascular medications included antihypertensives, lipid‐lowering drugs, diuretics, antiplatelets, ACE inhibitors, and related prescriptions; metabolic medications included any antidiabetic drugs such as metformin; respiratory medications were defined as prescribed asthma or COPD treatments; pain and anti‐inflammatory medications included prescribed analgesics and non‐steroidal anti‐inflammatory drugs; gastrointestinal medications consisted of proton pump inhibitors; mental health medications included antidepressants, antipsychotics, hypnotics, and other prescribed psychiatric drugs; and anti‐infectives comprised prescribed antibacterial agents. For each grouping, a binary indicator was generated to reflect whether at least one medication in that category was reported in the past week.

In addition, an overall medication burden measure was derived by summing the total number of distinct prescribed medications taken in the past 7 days. This was categorized as none, one, two, or three or more medications, with the latter representing polypharmacy. By combining overall burden with therapeutic‐specific groupings, the analysis captured both the extent of medication burden and the distribution of prescribing across major therapeutic domains.

### 2.3 Exposure

The exposure of interest was smoking and e‐cigarette status, derived from self‐reported survey items in the Health Survey for England 2021 on cigarette smoking and e‐cigarette use. These measures were combined to generate a four‐level categorical variable, with participants classified as follows:

- Never users – individuals who reported never smoking cigarettes and who had never tried e‐cigarettes.
- Current exclusive smokers – individuals who reported currently smoking cigarettes but not currently using e‐cigarettes.
- Current exclusive e‐cigarette users – individuals who reported currently using e‐cigarettes but not currently smoking cigarettes.
- Ex‐smokers – individuals who reported formerly smoking cigarettes but no longer do so, and are not currently using e‐cigarettes.

This approach ensured that each participant was assigned to a single, mutually exclusive category reflecting their present tobacco and nicotine use status. Participants who reported currently smoking cigarettes and currently using e‐cigarettes (dual users) were excluded from the analytic sample because our focus was on mutually exclusive groups and the potentially small sample size.

### 2.4 Covariates

Analyses adjusted for key sociodemographic factors that may confound the relationship between smoking or e‐cigarette use and prescribed medication burden. Age was categorized into five groups (16–34, 35–44, 45–54, 55–64, and ≥ 65 years) to capture life‐course variation in prescribing. Sex was included as a binary variable (male, female). Educational attainment was classified as degree or equivalent, higher education below degree level, secondary qualifications, or no formal qualifications. Area‐level socioeconomic deprivation was measured using quintiles of the 2019 Index of Multiple Deprivation (IMD), ranging from the least deprived to the most deprived quintile. These covariates were selected a priori because they are strongly associated with both tobacco and nicotine use patterns and healthcare utilization, including the likelihood of receiving prescriptions [27, 28, 29, 30]. Adjustment for these factors allowed us to better isolate the independent associations of smoking and e‐cigarette status with medication outcomes.

### 2.5 Statistical Analysis

We first conducted descriptive analyses to summarize participant characteristics and medication use across therapeutic categories by smoking and e‐cigarette status groups. Differences between groups were assessed using chi‐square tests for categorical variables.

The first outcome measure was therapeutic medication categories, represented as binary indicators of whether participants had received at least one prescription in each of the following domains: cardiovascular, metabolic, respiratory, pain and anti‐inflammatory, gastrointestinal, mental health, and anti‐infective medications. These outcomes were analysed using Firth penalized logistic regression to obtain adjusted ORs and 95% CIs, an approach chosen to minimize small‐sample bias in groups with low prevalence [31]. All models were adjusted for age group, sex, educational attainment, and area‐level deprivation quintile. The second outcome measure was overall medication burden, defined as the total number of prescribed medications taken in the past 7 days. This was categorized into four levels: none, one, two, and three or more medications, with the latter representing polypharmacy. To analyse this ordinal outcome, we used generalized ordered logistic regression (gologit2), which relaxes the proportional odds assumption and models the likelihood of being in higher medication categories. Odds ratios (ORs) and 95% confidence intervals (CIs) were reported, with never users as the reference group.

In addition, we conducted supplementary analyses directly comparing exclusive e‐cigarette users with exclusive cigarette smokers by re‐estimating all Firth penalized logistic regression models (for each therapeutic medication category) using exclusive smokers as the reference group and adjusting for age group, sex, educational attainment, and area‐level deprivation. We also re‐estimated the generalized ordered logistic regression model for overall medication burden with exclusive smokers as the reference group, adjusting for the same covariates.

Sensitivity analyses were conducted restricting the sample to participants aged 45 years and older, to reflect prescribing patterns more typical of midlife and older adults. In these models, we adjusted for sex, educational attainment, and area‐level deprivation. Analyses were performed using Stata version 18 (StataCorp, College Station, TX), and statistical significance was defined as *p* < 0.05 (two‐sided).

## 3 Results

In the analytic sample of 1657 adults who completed the nurse visit, never users formed the largest group (*n* = 1026), followed by ex‐smokers (*n* = 444), current smokers (*n* = 138), and current exclusive e‐cigarette users (*n* = 49) (Table 1). Age distribution differed significantly across groups (*p* < 0.001). Among never users, 37.4% were aged 65 years or older, compared with 27.5% of current smokers, 16.3% of current e‐cigarette users, and 55.2% of ex‐smokers. At the younger end, 24.5% of current e‐cigarette users were aged 35–44, compared with 14.5% of never users. Sex also varied by group (*p* = 0.016): males represented 41.6% of never users, 47.8% of current smokers, 51.0% of current e‐cigarette users, and 50.0% of ex‐smokers. Educational attainment showed pronounced gradients (*p* < 0.001). A degree or equivalent was most common among never users (40.6%) and least common among current smokers (15.9%), whereas having no qualifications was most frequent among current smokers (31.9%) and ex‐smokers (25.2%). Area‐level deprivation also showed clear inequalities (*p* < 0.001): 28.8% of never users lived in the least deprived quintile, compared with 14.5% of current smokers; conversely, 29.0% of current smokers and 16.3% of current e‐cigarette users lived in the most deprived quintile, compared with 10.4% of never users and 11.9% of ex‐smokers. Medication burden differed substantially between groups (*p* < 0.001). Overall, 44.7% of participants reported no prescribed medications, ranging from 50.7% of current smokers and 44.9% of current e‐cigarette users to only 33.1% of ex‐smokers. Use of three or more medications was highest among ex‐smokers (20.3%), compared with 8.6% of never users, 10.9% of current smokers, and 12.2% of current e‐cigarette users.

**Table 1:** Baseline Characteristics of Participants by Smoking and E‐Cigarette Status (*n* = 1657).

Characteristic	Never users (*n* = 1026)	Current smokers (*n* = 138)	Current e‐cigarette users (*n* = 49)	Ex‐smokers (*n* = 444)	Total (*n* = 1657)	*p*‐value
Age group, *n* (%)						< 0.001
16–34	123 (12.0)	17 (12.3)	6 (12.2)	25 (5.6)	171 (10.3)	
35–44	149 (14.5)	25 (18.1)	12 (24.5)	33 (7.4)	219 (13.2)	
45–54	170 (16.6)	19 (13.8)	9 (18.4)	54 (12.2)	252 (15.2)	
55–64	200 (19.5)	39 (28.3)	14 (28.6)	87 (19.6)	340 (20.5)	
65+	384 (37.4)	38 (27.5)	8 (16.3)	245 (55.2)	675 (40.7)	
Sex, *n* (%)						0.016
Male	427 (41.6)	66 (47.8)	25 (51.0)	222 (50.0)	740 (44.7)	
Female	599 (58.4)	72 (52.2)	24 (49.0)	222 (50.0)	917 (55.3)	
Education, *n* (%)						< 0.001
Degree or equivalent	417 (40.6)	22 (15.9)	13 (26.5)	113 (25.5)	565 (34.1)	
Higher education below degree	136 (13.3)	17 (12.3)	11 (22.5)	59 (13.3)	223 (13.5)	
Secondary	327 (31.9)	55 (39.9)	18 (36.7)	160 (36.0)	560 (33.8)	
No qualification	146 (14.2)	44 (31.9)	7 (14.3)	112 (25.2)	309 (18.6)	
Deprivation quintile, *n* (%)						< 0.001
Least deprived	295 (28.8)	20 (14.5)	12 (24.5)	112 (25.2)	439 (26.5)	
2	258 (25.2)	19 (13.8)	9 (18.4)	109 (24.6)	395 (23.8)	
3	216 (21.1)	29 (21.0)	9 (18.4)	83 (18.7)	337 (20.3)	
4	150 (14.6)	30 (21.7)	11 (22.5)	87 (19.6)	278 (16.8)	
Most deprived	107 (10.4)	40 (29.0)	8 (16.3)	53 (11.9)	208 (12.6)	
Medication burden, *n* (%)						< 0.001
None	501 (48.8)	70 (50.7)	22 (44.9)	147 (33.1)	740 (44.7)	
1	282 (27.5)	35 (25.4)	12 (24.5)	127 (28.6)	456 (27.5)	
2	155 (15.1)	18 (13.0)	9 (18.4)	80 (18.0)	262 (15.8)	
3+	88 (8.6)	15 (10.9)	6 (12.2)	90 (20.3)	199 (12.0)	

With respect to e‐cigarette history, 49 participants (3.0%) were classified as current exclusive e‐cigarette users. Among these, 47 (95.9%) reported a history of cigarette smoking, and 44 (89.8%) were former regular smokers, while only 2 (4.1%) had never smoked cigarettes. A further 116 participants (7.0%) had tried e‐cigarettes but were not current users; of these, 72 (62.1%) were current smokers and 44 (37.9%) were ex‐smokers, and none were never smokers.

Table 2 reports medication use by smoking and e‐cigarette status. Cardiovascular medication use was most frequent among ex‐smokers (48.7%) compared with never users (33.6%), current smokers (30.4%), and current e‐cigarette users (32.7%) (*p* < 0.001). Metabolic medication use followed a similar pattern, reported by 10.6% of ex‐smokers, 6.5% of never users, 2.2% of smokers, and 4.1% of e‐cigarette users (*p* = 0.003). Respiratory medication use was 11.7% among ex‐smokers, 11.6% among smokers, 12.2% among e‐cigarette users, and 6.7% among never users (*p* = 0.006). Use of pain and anti‐inflammatory medication was highest in e‐cigarette users (20.4%) and ex‐smokers (14.0%) compared with 8.6% of never users and 11.6% of smokers (*p* = 0.002). Gastrointestinal medication use was also more common among ex‐smokers (26.8%) than in never users (15.1%), smokers (13.8%), or e‐cigarette users (18.4%) (*p* < 0.001). Mental health medication use did not differ significantly between groups (*p* = 0.211), ranging from 15.7% in never users to 22.4% in e‐cigarette users. Anti‐infective use was uncommon overall but was reported by 4.7% of ex‐smokers compared with 1.3% of never users, none of the smokers, and 2.0% of e‐cigarette users (*p* < 0.001).

**Table 2:** Medication Use by Smoking and E‐Cigarette Status (*n* = 1657). Data are presented as *n* (column %).

Medication category	Never users (*n* = 1026)	Current smokers (*n* = 138)	Current e‐cigarette users (*n* = 49)	Ex‐smokers (*n* = 444)	Total (*n* = 1657)	*p*‐value*
Cardiovascular meds	345 (33.6)	42 (30.4)	16 (32.7)	216 (48.7)	619 (37.4)	< 0.001
Metabolic meds	67 (6.5)	3 (2.2)	2 (4.1)	47 (10.6)	119 (7.2)	0.003
Respiratory meds	69 (6.7)	16 (11.6)	6 (12.2)	52 (11.7)	143 (8.6)	0.006
Pain/anti‐inflammatory meds	88 (8.6)	16 (11.6)	10 (20.4)	62 (14.0)	176 (10.6)	0.002
Gastrointestinal meds	155 (15.1)	19 (13.8)	9 (18.4)	119 (26.8)	302 (18.2)	< 0.001
Mental health meds	161 (15.7)	30 (21.7)	11 (22.4)	77 (17.3)	279 (16.8)	0.211
Anti‐infectives	13 (1.3)	0 (0.0)	1 (2.0)	21 (4.7)	35 (2.1)	< 0.001

Cardiovascular meds: antihypertensives, lipid‐lowering drugs, diuretics, antiplatelets, and ACE inhibitors.

Metabolic meds: antidiabetic drugs including metformin.

Respiratory meds: prescribed asthma or COPD medications.

Pain/anti‐inflammatory meds: prescribed analgesics and NSAIDs.

Gastrointestinal meds: proton pump inhibitors.

Mental health meds: antidepressants, antipsychotics, hypnotics, or other prescribed psychiatric drugs.

Anti‐infectives: prescribed antibacterial medications.

* *P*‐values from chi‐square tests.

Table 3 reports adjusted associations between smoking/e‐cigarette status and prescribed medication use, using never users as the reference group. For cardiovascular medications, no significant differences were observed for current smokers (OR = 0.80, 95% CI: 0.50–1.29, *p* = 0.36), current e‐cigarette users (OR = 1.68, 95% CI: 0.82–3.42, *p* = 0.15), or ex‐smokers (OR = 1.18, 95% CI: 0.90–1.54, *p* = 0.24). Current smokers had significantly lower odds of metabolic medication use (OR = 0.28, 95% CI: 0.09–0.86, *p* = 0.03), while no significant associations were found for e‐cigarette users (OR = 0.93, 95% CI: 0.25–3.47, *p* = 0.92) or ex‐smokers (OR = 1.28, 95% CI: 0.85–1.92, *p* = 0.24).

**Table 3:** Adjusted Odds Ratios (95% CI) and *p*‐values for Medication Use by Smoking/E‐Cigarette Status.

Medication category	Current smokers OR (95% CI), *p*‐value	Current e‐cigarette users OR (95% CI), *p*‐value	Ex‐smokers OR (95% CI), *p*‐value
Cardiovascular meds	0.80 (0.50–1.29), *p* = 0.36	1.68 (0.82–3.42), *p* = 0.15	1.18 (0.90–1.54), *p* = 0.24
Metabolic meds	**0.28 (0.09–0.86), *p*** = **0.03**	0.93 (0.25–3.47), *p* = 0.92	1.28 (0.85–1.92), *p* = 0.24
Respiratory meds	1.57 (0.86–2.86), *p* = 0.14	2.24 (0.93–5.42), *p* = 0.07	**1.72 (1.17–2.55), *p*** = **0.006**
Pain/anti‐inflammatory meds	1.28 (0.71–2.32), *p* = 0.41	**3.61 (1.70–7.67), *p*** = **0.001**	1.33 (0.93–1.90), *p* = 0.12
Gastrointestinal meds	0.84 (0.49–1.43), *p* = 0.52	1.69 (0.78–3.62), *p* = 0.18	**1.56 (1.17–2.07), *p*** = **0.002**
Mental health meds	1.28 (0.81–2.02), *p* = 0.29	1.54 (0.77–3.09), *p* = 0.22	1.12 (0.83–1.53), *p* = 0.46
Anti‐infectives	‐‐‐	2.47 (0.43–14.35), *p* = 0.31	**3.32 (1.63–6.79), *p*** = **0.001**

*P*‐values from Firth penalized logistic regression models adjusted for age group, sex, education, and area‐level deprivation (quintiles of the Index of Multiple Deprivation 2019).

Reference group = never users.

Cardiovascular meds: antihypertensives, lipid‐lowering drugs, diuretics, antiplatelets, and ACE inhibitors.

Metabolic meds: antidiabetic drugs including metformin.

Respiratory meds: prescribed asthma or COPD medications.

Pain/anti‐inflammatory meds: prescribed analgesics and NSAIDs.

Gastrointestinal meds: proton pump inhibitors. Mental health meds: antidepressants, antipsychotics, hypnotics, or other prescribed psychiatric drugs.

Anti‐infectives: prescribed antibacterial medications.

Models with zero events in a category (e.g., anti‐infective use among current smokers) are shown as “–” because estimates are not statistically reliable despite Firth penalization.

For respiratory medications, higher odds were observed among ex‐smokers (OR = 1.72, 95% CI: 1.17–2.55, *p* = 0.006), whereas estimates for current smokers (OR = 1.57, 95% CI: 0.86–2.86, *p* = 0.14) and e‐cigarette users (OR = 2.24, 95% CI: 0.93–5.42, *p* = 0.07) did not reach significance. Pain and anti‐inflammatory medication use were significantly higher among current e‐cigarette users (OR = 3.61, 95% CI: 1.70–7.67, *p* = 0.001). Ex‐smokers had non‐significantly higher odds (OR = 1.33, 95% CI: 0.93–1.90, *p* = 0.12), while no difference was observed for current smokers (OR = 1.28, 95% CI: 0.71–2.32, *p* = 0.41).

Ex‐smokers also had significantly greater odds of gastrointestinal medication use (OR = 1.56, 95% CI: 1.17–2.07, *p* = 0.002), while no associations were found for current smokers (OR = 0.84, 95% CI: 0.49–1.43, *p* = 0.52) or e‐cigarette users (OR = 1.69, 95% CI: 0.78–3.62, *p* = 0.18). No significant associations were identified for mental health medications across groups: ORs ranged from 1.12 among ex‐smokers to 1.54 among e‐cigarette users, with all *p* > 0.20.

For anti‐infectives, ex‐smokers had markedly higher odds (OR = 3.32, 95% CI: 1.63–6.79, *p* = 0.001), whereas the estimates for e‐cigarette users (OR = 2.47, 95% CI: 0.43–14.35, *p* = 0.31) were imprecise and not statistically significant, and no reliable estimate was available for current smokers due to zero events.

**Figure 1:**
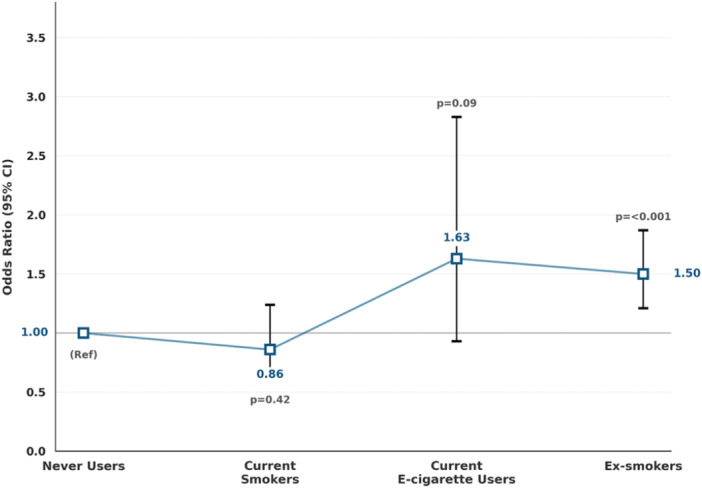
Adjusted Associations Between Smoking/E‐Cigarette Status and Medication Burden. Models adjusted for age group, sex, education, and area‐level deprivation (IMD 2019 quintiles). Medication burden is categorized as none, 1, 2, and 3+ prescribed medications. Odds ratios represent the odds of being in a higher burden category at each threshold. Higher medication burden is defined as being in a more severe category on the ordinal scale, ranging from no medications to one medication, two medications, and three or more medications. Results estimated using generalized ordered logistic regression (gologit2), which relaxes the proportional odds assumption violated in standard ordinal logistic regression.

Figure 1 shows the adjusted associations between smoking/e‐cigarette status and overall medication burden from the generalized ordered logistic regression. Never users were the reference group. Compared with never users, current smokers had similar odds of higher medication burden (OR = 0.86, 95% CI: 0.60–1.24, *p* = 0.42). Current e‐cigarette users had a non‐significant trend toward higher burden (OR = 1.63, 95% CI: 0.93–2.83, *p* = 0.09). Ex‐smokers, however, had significantly greater odds of reporting higher medication burden (OR = 1.50, 95% CI: 1.21–1.87, *p* < 0.001).

### 3.1 Supplementary Analyses

In supplementary analyses directly comparing exclusive e‐cigarette users with exclusive cigarette smokers, we found no statistically significant differences across most therapeutic medication categories. The only exception was pain and anti‐inflammatory medications, where exclusive e‐cigarette users had higher odds of use compared with exclusive smokers (OR = 2.82; 95% CI 1.16–6.88; *p* = 0.023). In supplementary ordinal regression analyses examining overall medication burden, exclusive e‐cigarette users also showed higher cumulative odds of being in a higher medication burden category relative to exclusive smokers (OR = 1.88; 95% CI 1.00–3.53; *p* = 0.051), although statistical evidence was weak. This borderline pattern may reflect residual confounding by differences in underlying health status or smoking history not fully captured in the model (e.g., prior smoking intensity, duration, or time since cessation).

### 3.2 Sensitivity Analyses

In sensitivity analyses restricted to participants aged 45 years and older (*n* = 1267), findings were broadly consistent with the main results. Ex‐smokers showed higher odds of several individual medication categories, including cardiovascular (OR = 1.34, 95% CI 1.03–1.73, *p* = 0.027), respiratory (OR = 1.74, 95% CI 1.15–2.63, *p* = 0.009), pain/anti‐inflammatory (OR = 1.48, 95% CI 1.02–2.14, *p* = 0.038), gastrointestinal (OR = 1.68, 95% CI 1.26–2.25, *p* < 0.001), and anti‐infective medications (OR = 4.09, 95% CI 1.87–8.94, *p* < 0.001). Current e‐cigarette users had elevated odds of pain/anti‐inflammatory medication use (OR = 3.85, 95% CI 1.64–9.06, *p* = 0.002), but associations with other categories were not significant. Current smokers showed no clear differences compared with never users across medication types. Analysis of medication burden using generalized ordered logistic regression confirmed that ex‐smokers were more likely to report higher prescribing levels (OR = 1.68, 95% CI 1.34–2.12, *p* < 0.001). By contrast, both current smokers and current e‐cigarette users did not differ significantly from never users.

## 4 Discussion

This study provides new evidence on the relationship between smoking, e‐cigarette use, and prescribed medication burden in a large, nationally representative sample of adults in England. We found substantial differences in both the total number of prescribed medications and the distribution of therapeutic categories across user groups. Ex‐smokers consistently reported the highest overall medication burden, with one in five prescribed three or more medicines, compared with fewer than one in ten never users. Current smokers did not differ significantly from never users in terms of overall burden, while current exclusive e‐cigarette users showed a non‐significant trend toward higher burden, occupying an intermediate position between never users and ex‐smokers. These findings suggest that smoking leaves a lasting legacy in terms of prescribing patterns, and that e‐cigarette users may follow a distinct trajectory that does not align fully with either smokers or never users. To our knowledge, this is the first study to directly compare prescribed medication burden across these four distinct groups, moving beyond the narrower lens of medication use to provide a fuller picture of how tobacco and nicotine use shape population prescribing.

Our results align with the well‐established evidence linking smoking to chronic disease and pharmacological treatment requirements [32, 33, 34]. Ex‐smokers reported higher use of cardiovascular, respiratory, gastrointestinal, and anti‐infective medications compared with never users, reflecting the long‐term health consequences of prior tobacco use [35]. Interestingly, current smokers did not differ significantly from never users in most categories and, in fact, showed lower odds of metabolic medication use. This pattern may partly reflect their younger age profile, selective patterns of healthcare engagement, and the so‐called “healthy smoker effect,” whereby smokers who remain in the population are those less affected by illness, while those who become unwell are more likely to quit [36, 37]. Ex‐smokers carried higher prescribing levels than current smokers across several categories, suggesting that the benefits of quitting in terms of future disease risk do not immediately translate into reduced medication needs. This is likely due to the cumulative impact of years of smoking, which results in chronic conditions that persist even after cessation [38, 39, 40]. However, reverse causation is also possible, as individuals may quit smoking after developing illness and requiring treatment, thereby contributing to the higher prescribing levels observed among ex‐smokers. The persistence of higher medication burden among ex‐smokers reinforces the importance of early smoking prevention and cessation to reduce long‐term healthcare demand. It is also likely that the ex‐smoker group is heterogeneous, encompassing individuals who quit recently as well as those who stopped many years earlier and who may or may not have used e‐cigarettes during their transition away from smoking. We were unable to examine quit duration or transition pathways in detail because of data and sample size constraints, and this remains an important area for future research.

The position of current exclusive e‐cigarette users warrants particular attention. Although the group was smaller in size, we observed higher odds of pain and anti‐inflammatory prescribing compared with never users. In supplementary analyses directly comparing exclusive e‐cigarette users with exclusive smokers, exclusive e‐cigarette users also showed higher odds of pain and anti‐inflammatory medication use and higher cumulative odds of being in a higher overall medication burden category, although the latter did not reach conventional statistical significance. Descriptive analyses showed that almost all current exclusive e‐cigarette users were former smokers, the majority of whom had previously smoked regularly, supporting the interpretation that their prescribing burden largely reflects the legacy of prior smoking rather than de novo effects of vaping. At the same time, overall medication burden showed only a non‐significant trend toward being greater. Very little is known about prescribing burden in this group, as previous studies have largely focused on disease incidence, quality of life, or self‐reported health. Our results suggest that, at a population level, exclusive e‐cigarette users do not fully resemble either never users or current smokers, instead reflecting an intermediate profile shaped by prior smoking. These findings highlight the importance of ongoing monitoring as e‐cigarette uptake grows, to clarify long‐term implications for prescribing and healthcare demand.

The social patterning of medication burden observed in this study adds another layer of complexity. Both smoking and prescribing are more common in deprived areas, and even after adjusting for education, sex, and area‐level deprivation, differences in burden across user groups remained marked. This suggests that smoking and e‐cigarette behaviors are associated with differences in prescribing that are not fully explained by these measured sociodemographic factors, although unmeasured aspects of socioeconomic position may still play an important role. Previous work has documented socioeconomic disparities in smoking prevalence and in multimorbidity [41, 42, 43, 44, 45], but few studies have examined their joint contribution to medication burden. By highlighting these associations, our findings underline the importance of addressing tobacco and nicotine use not only as a behavioral risk factor but also as a driver of inequalities in healthcare utilization.

The implications of these findings extend to both clinical practice and public health policy. For clinicians and pharmacists, recognizing that ex‐smokers carry a disproportionate medication burden highlights the need for proactive medication reviews and optimization in this group, with particular attention to cardiovascular, respiratory, and gastrointestinal medicines. For public health, the results reinforce the benefits of smoking prevention and early cessation in reducing long‐term prescribing demand, while also pointing to the need to monitor the evolving role of e‐cigarettes in shaping health system use. By framing outcomes in terms of medication burden, our study connects tobacco control with prescribing practices and healthcare planning, offering a novel perspective on the wider impacts of smoking behaviors and nicotine use. This has potential policy relevance in terms of workforce planning for pharmacy services, resource allocation in primary care, and cost forecasting in health systems.

This study has several strengths and limitations that should be considered. A key strength is the use of nurse‐recorded prescribed medication data, which reduces reliance on unaided self‐report and is likely to minimize recall error compared with questionnaire‐only reporting. The nationally representative sampling framework of the Health Survey for England supports external validity, and the use of two complementary outcomes, overall medication burden and therapeutic categories, provides a broader picture of prescribing patterns. The inclusion of e‐cigarette use status as a distinct exposure group adds novelty to the evidence base. However, several limitations apply. First, the cross‐sectional design precludes causal inference. Second, medication use was assessed over the previous 7 days. While this short recall window minimizes recall bias, it may not adequately capture chronic medicines taken intermittently or treatments used outside the assessment period, and therefore may underestimate longer‐term medication burden for some participants. Third, analyses were restricted to participants who completed a nurse visit, which may introduce selection bias if nurse‐visit attenders differ systematically from non‐attenders in underlying health status, healthcare engagement, or medication use. Fourth, the relatively small number of current e‐cigarette users limited statistical power for some therapeutic categories, and we could not assess prescription appropriateness, dosage, adherence, or over‐the‐counter medicines. Although we adjusted for key sociodemographic characteristics and smoking status, residual confounding by unmeasured health conditions remains possible. These limitations should be considered when interpreting the observed differences in medication burden and therapeutic categories across smoking and e‐cigarette use groups.

Future studies should build on these findings by using longitudinal designs to track prescribing trajectories over time, clarifying how medication burden evolves as individuals transition between smoking, e‐cigarette use, and cessation. Larger and better‐characterized samples of exclusive e‐cigarette users are needed to more precisely define their prescribing patterns and to disentangle the legacy effects of prior smoking from any independent impact of vaping. Research incorporating information on medication appropriateness, dosage, and over‐the‐counter use would also provide a richer understanding of the actual burden of treatment. In addition, qualitative studies could explore patient and practitioner perspectives on how smoking history shapes prescribing decisions, while health economic analyses could estimate the costs associated with smoking‐ and cessation‐related medication burden. Together, these approaches would offer a more comprehensive assessment of the long‐term implications of tobacco and nicotine behaviors for prescribing practices and healthcare systems.

## 5 Conclusion

This study provides population‐based evidence describing how prescribed medication burden varies across smoking and e‐cigarette status groups. Ex‐smokers carried the highest burden, reflecting the enduring health consequences of prior tobacco use, whereas current smokers did not differ significantly from never users after adjustment. Exclusive e‐cigarette users showed a distinct, intermediate prescribing profile, with higher use of pain and anti‐inflammatory medications, although this finding was based on a small subgroup and should be interpreted cautiously. These results indicate that smoking behaviors and nicotine use are associated with differences in prescribing at the population level over and above age, sex, education, and area‐level deprivation, but broader socioeconomic factors may still contribute to these patterns. By framing outcomes in terms of medication burden rather than disease endpoints alone, this study offers a novel perspective on the wider impacts of tobacco and nicotine use on healthcare demand. Further longitudinal research is needed to clarify how medication burden evolves as individuals transition between smoking, e‐cigarette use, and cessation, and to disentangle the legacy effects of prior smoking from any independent impact of vaping.

## Author Contributions

Yusuff Adebayo Adebisi conceived the study, conducted the data analysis, interpreted the results, and drafted the first version of the manuscript. Leonard Ighodalo Uzairue and Rachael Oluwatoyosi Farayola contributed to the interpretation of findings and manuscript drafting. Eseosa Iyagbaye and Christian Joseph N. Ong contributed to the literature review, contextualisation of findings, and manuscript editing. Akpevwe Emmanuella Benson and Adeyemo Deborah Temitope contributed to reference management, data curation, and manuscript revision. Ngoni Veddie Muzondo contributed to methodological input, statistical review, and critical revision of the manuscript. All authors reviewed and approved the final version of the manuscript and agree to be accountable for all aspects of the work.

## Ethics Statement

This study is a secondary analysis of the Health Survey for England (HSE) 2021 data, and no additional ethical approval was required. The original HSE 2021 study received approval from the East Midlands – Nottingham 2 Research Ethics Committee (Reference no. 15/EM/0254). All procedures were conducted in accordance with the ethical standards of the responsible committee on human experimentation and with the principles of the Declaration of Helsinki.

## Consent

All participants in the Health Survey for England (HSE) 2021 provided informed consent prior to data collection. Consent procedures were approved by the East Midlands – Nottingham 2 Research Ethics Committee (Reference no. 15/EM/0254). As this study involved secondary analysis of anonymised survey data, no additional consent was required.

## Conflicts of Interest

The authors declare that they have no known competing financial interests or personal relationships that could have appeared to influence the work reported in this paper. However, Yusuff Adebayo Adebisi has previously received funding through the Tobacco Harm Reduction Scholarship and the Kevin Molloy Fellowship, both awarded by Knowledge‐Action‐Change (KAC), an independent public health organization based in the UK. KAC is funded by Global Action to End Smoking (GA), an independent US non‐profit 501(c)(3) grant‐making organization that supports health and science research, cessation education, and agricultural transformation initiatives.

## Transparency Statement

The lead author Yusuff Adebayo Adebisi affirms that this manuscript is an honest, accurate, and transparent account of the study being reported; that no important aspects of the study have been omitted; and that any discrepancies from the study as planned (and, if relevant, registered) have been explained.

## Data Availability

The Health Survey for England 2021 datasets are available from the UK Data Service: End User Licence (SN 9319) and Special Licence (SN 9320, DOI: 10.5255/UKDA‐SN‐9320‐1).
